# Molecular epidemiology and antimicrobial resistance profiles of *Clostridioides difficile* in Xi’an

**DOI:** 10.3389/fmicb.2026.1882279

**Published:** 2026-07-01

**Authors:** Yang Luan, Yiting Yang, Yixian Tian, He Zhao, Hao Li, Huan Wang, Qingyu Wang, Xiaoli Wei, Baozhong Chen

**Affiliations:** Xi’an Municipal Center for Disease Control and Prevention, Xi'an, Shaanxi, China

**Keywords:** antimicrobial resistance, *Clostridioides difficile*, multilocus sequence typing, PCR-ribotyping, whole-genome sequencing

## Abstract

**Background:**

Clostridioides difficile is a leading cause of antibiotic-associated diarrhoea and a growing public health concern, yet data on its genomic characteristics and antimicrobial resistance in Northwest China remain scarce.

**Methods:**

This study investigated the molecular epidemiology and resistance profiles of C. difficile in Xi’an using 149 toxigenic isolates recovered from diarrhoeal stool samples collected across seven hospitals between 2018 and 2025. Isolates were characterised by whole-genome sequencing (WGS), multilocus sequence typing (MLST), and PCR-ribotyping, with antimicrobial susceptibility determined by the Etest method.

**Results:**

The predominant toxin profile was A+B+CDT− (88.6%, 132/149), followed by A−B+CDT− (8.7%, 13/149) and A+B+CDT+ (2.7%, 4/149); binary toxin (CDT)-positive isolates were exclusively A+B+. MLST and ribotyping resolved 29 sequence types (STs) and 48 ribotypes (RTs), with ST42/RT106 (17.4%), ST3/RT001 (9.4%), and ST54/RT012 (8.7%) the most prevalent. All isolates remained susceptible to metronidazole and vancomycin, whereas high resistance proportions were observed for ciprofloxacin (84.6%), erythromycin (56.4%), and clindamycin (53.7%). The Thr82Ile substitution in GyrA was detected in fluoroquinolone-resistant isolates, and the Arg505Lys substitution in RpoB in rifampicin-resistant isolates.

**Conclusion:**

These findings establish, to our knowledge, the first comprehensive genomic profile of C. difficile in Xi’an and may inform regional surveillance and antimicrobial stewardship efforts.

## 1 Introduction

*Clostridioides difficile* is an obligately anaerobic, Gram-positive, spore-forming bacillus and the most common cause of antibiotic-associated diarrhoea (AAD) and healthcare-associated intestinal infection (Leffler and Lamont, 2015). *C. difficile* infection (CDI) ranges from mild, self-limiting diarrhoea to life-threatening pseudomembranous colitis, toxic megacolon, and death (Vazquez-Cuesta et al., 2023; Czepiel et al., 2019). Two exotoxins—enterotoxin A (TcdA) and cytotoxin B (TcdB), encoded by *tcdA* and *tcdB*—account for most of its pathogenicity (Janezic et al., 2020); a subset of strains also harbour the binary toxin (CDT) genes *cdtA* and *cdtB*.

The hypervirulent BI/NAP1/027 strain (ribotype 027) has driven CDI outbreaks across Europe and North America since 2003 (McDonald et al., 2005). RT027 reached mainland China in 2012 and has since been tied to hospital outbreaks there (Jia et al., 2016; Zhou et al., 2014), with molecular characterisation confirming its presence in major Chinese hospitals (Zhang et al., 2021). Alongside rising CDI incidence, antimicrobial resistance in *C. difficile* has been creeping upward. Metronidazole and vancomycin, the mainstay CDI treatments, are losing susceptibility in some settings (Pelaez et al., 2002; Baines et al., 2008), and resistance to erythromycin, clindamycin, and fluoroquinolones is already widespread among Chinese isolates (Dang et al., 2024; Lv et al., 2022). Knowing the local susceptibility picture is therefore important—not only for flagging emerging epidemic clones, but for understanding which endemic strains persist in a given region. Such surveillance also depends on reliable typing tools.

Molecular typing is central to CDI outbreak investigation and control. PCR-ribotyping, which reads length polymorphisms (200–600 bp) in the 16S–23S rRNA intergenic spacer, remains the workhorse method in Europe and the United States (Emele et al., 2019; Calderaro et al., 2022). Multilocus sequence typing (MLST), based on seven housekeeping loci, yields reproducible, portable data that are straightforward to compare across laboratories (Kamboj et al., 2021). Whole-genome sequencing (WGS) goes further, capturing genomic features, phylogenetic relationships, resistance determinants (Saldanha et al., 2020; Knight et al., 2015), and toxin variants (Li et al., 2020) in a single run. With sequencing costs continuing to drop, WGS is moving from a research tool towards routine use for tracking *C. difficile* evolution and transmission.

*C. difficile* has been little studied in Northwest China, and Xi’an, the region’s largest city, has been particularly under-studied. In this study, we surveyed the prevalence, toxin genotypes, and antimicrobial resistance of *C. difficile* across seven hospitals in Xi’an. Isolates from diarrhoeal faecal samples were characterised by MLST, PCR-ribotyping, and WGS, and further assessed for antimicrobial susceptibility, resistance-associated mutations, and virulence gene carriage.

## 2 Materials and methods

### 2.1 Study setting, sample collection and *Clostridioides difficile* isolation

#### 2.1.1 Study setting

Unlinked, non-duplicate diarrhoeal stool specimens were prospectively collected from seven hospitals in Xi’an, the capital city of Shaanxi Province and the largest city in Northwest China (population: ~13 million). The participating sites comprised five tertiary-care (Grade A) general hospitals, one tertiary-care paediatric hospital, and one provincial geriatric hospital, collectively covering central, eastern, western, southern, and northern districts of the city. Detailed characteristics of each hospital are provided in Supplementary Table S3.

#### 2.1.2 Sample collection, culture and identification

Stool specimens were collected from hospitalised patients who developed diarrhoea (≥3 unformed stools within 24 h) at least 48 h after admission across the seven participating hospitals between October 2018 and December 2025. All specimens were transported to the Xi’an Municipal Center for Disease Control and Prevention for processing. For alcohol shock, stool was mixed with an equal volume of absolute ethanol, vortexed, and left at room temperature for 30 min. After centrifugation at 3,000 × g for 10 min, the pellet was inoculated onto cefoxitin–cycloserine–fructose agar (CCFA; CM0601 supplemented with SR0096, Oxoid, Basingstoke, UK) and incubated in an intelligent anaerobic culture system (DW-100A-K, DW Microbiology, Hangzhou, China) at 37 °C for 48 h. Putative *C. difficile* colonies were identified by characteristic colony morphology, Gram stain, and odour, and confirmed by MALDI-TOF mass spectrometry (AUTOF MS 2000, Autobio Diagnostics, Zhengzhou, China). All confirmed isolates were stored in 20% glycerol broth at −80 °C pending further analysis.

#### 2.1.3 Impact of COVID-19 on sampling

A notable decrease in sample volume was observed in 2020 (61 stool specimens yielding 8 isolates; Table 1). This reduction coincided with the COVID-19 pandemic, during which healthcare-seeking behaviour for non-COVID conditions declined markedly, hospital admissions for diarrhoeal illness decreased, and enhanced infection control measures—including mandatory masking, environmental disinfection, and visitor restrictions—may have concurrently reduced healthcare-associated *C. difficile* transmission. Importantly, no changes were made to the stool collection, processing, or culture methodology throughout the entire seven-year study period.

**Table 1:** *C. difficile* toxin gene and patient demographics.

Years	Isolation proportion (CD/No. samples)	Toxin genotype				Gender		Age			
		A+B+CDT−	A−B+CDT−	A+B+CDT+	Toxigenic isolates	Male	Female	≤5 years	6–18 years	19–65 years	≥65 years
2018–2019	12.5% (22/176)	13	2	1	16	12	4	1	0	8	7
2020	13.1% (8/61)	5	2	0	7	4	3	0	0	3	4
2021	10.1% (19/188)	11	2	0	13	8	5	0	0	4	9
2022	9.0% (39/434)	23	5	1	29	14	15	12	2	4	11
2023	7.7% (32/413)	22	1	1	24	11	13	12	1	1	10
2024	6.7% (32/479)	22	1	0	23	11	12	7	6	4	6
2025	7.3% (43/590)	36	0	1	37	26	11	19	3	9	6
Total	8.3% (195/2341)	132 (88.6%)	13 (8.7%)	4 (2.7%)	149	86	63	51	12	33	53

### 2.2 Detection of toxin genes and PCR-ribotyping

Total genomic DNA was extracted from bacterial cells using a DNA Mini-Kit (51304, Qiagen, Hilden, Germany) according to the manufacturer’s instructions. The toxin genes *tcdA*, *tcdB*, and *cdtA*/*cdtB* were detected by multiplex PCR following established protocols (Zhang et al., 2020). PCR-based ribotyping was carried out using the capillary gel electrophoresis protocol originally described by Stubbs et al. (1999). The primers 16S-F (5′-GTGCGGCTGGATCACCTCCT-3′) and 23S-R (5′-CCCTGCACCCTTAATAACTTGACC-3′) were used for amplification; the 16S rRNA gene primer was labelled at the 5′ end with carboxyfluorescein. PCR fragments were analysed on an ABI 3730 genetic analyser (Applied Biosystems, Foster City, CA, United States) equipped with a 50 cm capillary loaded with POP7 gel (Applied Biosystems). Data were submitted to the WEBRIBO database^1^ for ribotype assignment.

### 2.3 Whole genome sequencing and bioinformatic analysis

Genomic sequencing was performed on the BGI DNBSEQ-G50 platform with 2 × 150 bp paired-end reads. Sequence data were processed and quality-controlled according to a standard pipeline (Zeng et al., 2026). Sequence types (STs), allele numbers, and clades were assigned using the *C. difficile* PubMLST database.^2^ Genetic relationships among isolates were visualised as a minimum-spanning tree using BioNumerics v7.6 (Applied Maths). Antimicrobial resistance (AMR) genes were predicted with ABRicate v1.0.1^3^ using default parameters against the Comprehensive Antibiotic Resistance Database (CARD) (Landman et al., 2024). Virulence factors were predicted in the same manner against the Virulence Factor Database (VFDB) (Landman et al., 2024). Resistance-associated substitutions in *gyrA*, *gyrB*, and *rpoB* were screened with Snippy v4.6.0 against the *C. difficile* 630 reference genome (accession number AM180355.1).

### 2.4 Core-genome SNP analysis

SNP analysis of all *C. difficile* genomes was performed with Snippy v4.6.0 using default parameters, with *C. difficile* 630 set as the reference strain. The core-genome alignment was filtered to remove recombination regions, and polymorphic sites were extracted using Gubbins v3.4.0 (Croucher et al., 2015). A maximum-likelihood phylogenetic tree was constructed in IQ-TREE v2.4.0 (Minh et al., 2020), with 1,000 ultrafast bootstrap replicates for branch support. Isolates were clustered into genetically distinct lineages by population structure inference using FastBAPS v1.0.8 (Tonkin-Hill et al., 2019). Tree visualisation and annotation were performed with the R packages ggplot2 (v4.0.1) (Wickham, 2016) and ggtree (v3.14.0) (Yu et al., 2016).

### 2.5 Antimicrobial susceptibility testing

Antimicrobial susceptibility of *C. difficile* isolates was determined by the Etest method against the following agents: metronidazole, vancomycin, rifampicin, levofloxacin, ciprofloxacin, meropenem, erythromycin, clindamycin, moxifloxacin, and tetracycline. Cultures were grown on Brucella agar (BBL BD, United States) supplemented with 5% defibrinated sheep blood, vitamin K (1 μg/mL), and haemin (5 μg/mL). Resistance breakpoints were adopted from the Clinical and Laboratory Standards Institute (CLSI) and the European Committee on Antimicrobial Susceptibility Testing (EUCAST) guidelines for anaerobic bacteria, as follows: metronidazole and vancomycin, ≥2 μg/mL; meropenem and tetracycline, ≥16 μg/mL; moxifloxacin and clindamycin, ≥8 μg/mL. Breakpoints for rifampicin (≥4 μg/mL), levofloxacin (≥8 μg/mL), erythromycin (≥8 μg/mL), and ciprofloxacin (≥8 μg/mL) were adopted from a previous study (Li et al., 2019). *C. difficile* ATCC 700057 served as the quality control reference strain throughout susceptibility testing.

### 2.6 Statistical analysis

Statistical analyses were performed with IBM SPSS Statistics v26 (IBM Corp., Armonk, NY, United States).

## 3 Results

### 3.1 Toxin profile and clinical characteristics

A total of 2,341 non-duplicate stool specimens were collected from patients with diarrhoea across seven hospitals over a seven-year period. From these, 195 (8.3%, 195/2,341) *C. difficile* isolates were cultured and identified. Among them, 149 (76.4%, 149/195) were toxigenic and 46 (23.6%, 46/195) were non-toxigenic. Of the 149 toxigenic isolates, 53 (35.6%, 53/149) were recovered from patients aged ≥65 years and 51 (34.2%, 51/149) from those aged ≤5 years. The toxigenic strains were classified into three toxin types: 132 A+B+CDT− strains (88.6%, 132/149), 13 A−B+CDT− strains (8.7%, 13/149), and 4 A+B+CDT+ strains (2.7%, 4/149) (Table 1).

### 3.2 Multi-locus sequence typing and PCR-ribotyping

**Figure 1:**
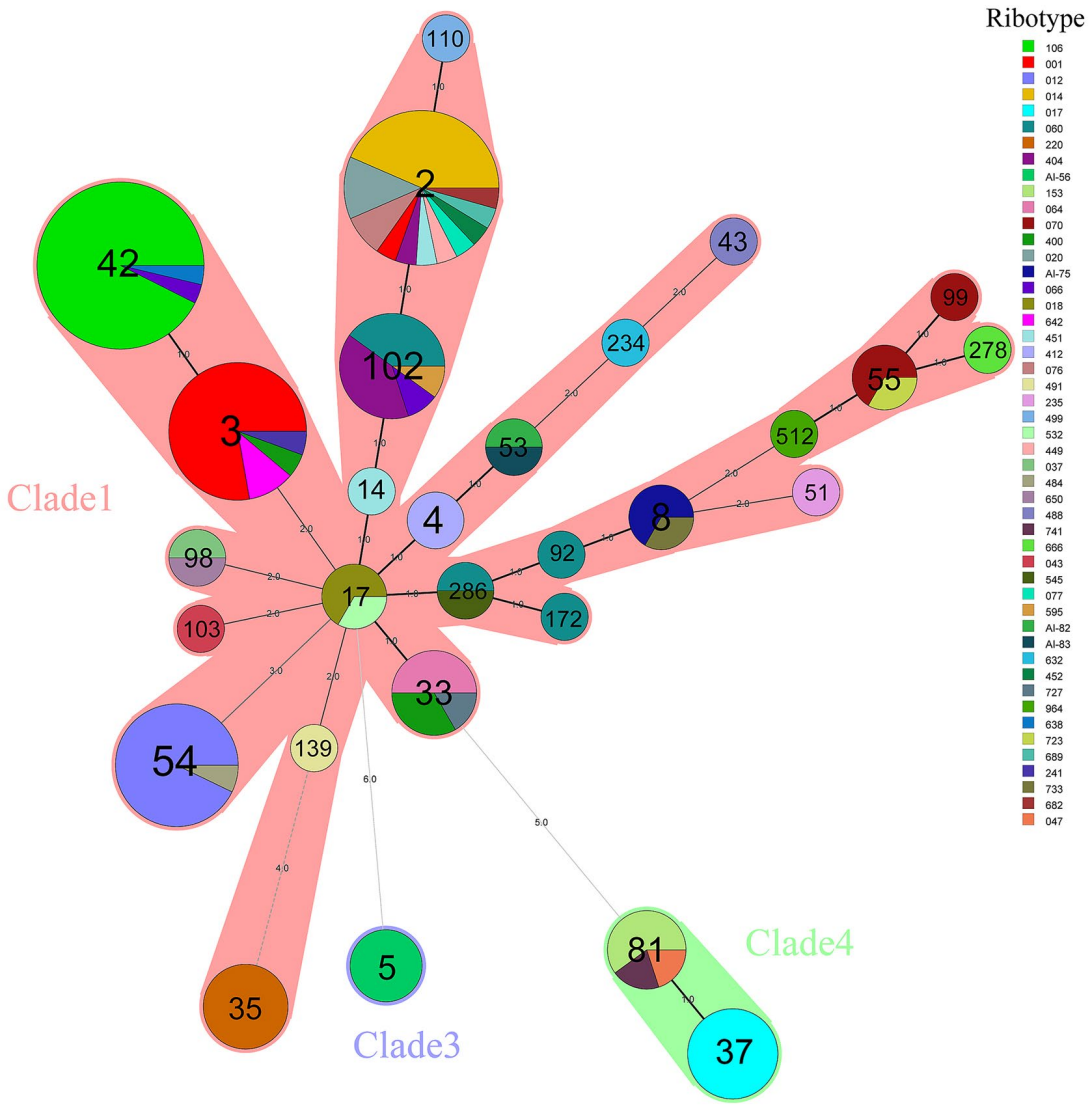
Minimum spanning tree based on multilocus sequence typing (MLST) allelic profiles. Each circle represents a sequence type (ST). The fill colour of a circle indicates PCR-ribotypes, and the border colour denotes MLST clades. Numbers on the connecting lines represent the number of allele differences between profiles.

MLST resolved 29 STs among the 149 toxigenic *C. difficile* isolates. The most prevalent ST was ST42 (18.1%, 27/149), followed by ST2 (15.4%, 23/149), ST3 (12.1%, 18/149), and ST54 (9.4%, 14/149). The remaining STs each accounted for <7% of isolates. Most isolates (89.3%, 133/149) belonged to Clade 1, with 8.1% (12/149) assigned to Clade 4 and only four isolates (2.7%, 4/149) to Clade 3 (Table 2; Figure 1).

**Table 2:** Multilocus sequence typing (MLST), ribotype and toxin genotypes of the 149 toxigenic *C. difficile* isolates.

MLST clade	MLST ST	Ribotype	Toxin gene	No. of isolates
Clade 1	ST2	001	A+B+CDT−	1
		014	A+B+CDT−	10
		020	A+B+CDT−	3
		076	A+B+CDT−	2
		077	A+B+CDT−	1
		404	A+B+CDT−	1
		449	A+B+CDT−	1
		451	A+B+CDT−	1
		452	A+B+CDT−	1
		682	A+B+CDT−	1
		689	A+B+CDT−	1
	ST3	001	A+B+CDT−	14
		241	A+B+CDT−	1
		400	A+B+CDT−	1
		642	A+B+CDT−	2
	ST4	412	A+B+CDT−	2
	ST8	AI-75	A+B+CDT−	2
		733	A+B+CDT−	1
	ST14	451	A+B+CDT−	1
	ST17	018	A+B+CDT−	2
		532	A−B+CDT−	1
	ST33	064	A+B+CDT−	3
		400	A+B+CDT−	2
		727	A+B+CDT−	1
	ST35	220	A+B+CDT−	6
	ST42	106	A+B+CDT−	26
		066	A+B+CDT−	1
	ST43	488	A+B+CDT−	1
	ST51	235	A+B+CDT−	1
	ST53	AI-82	A+B+CDT−	1
		AI-83	A+B+CDT−	1
	ST54	012	A+B+CDT−	13
		484	A+B+CDT−	1
	ST55	070	A+B+CDT−	2
		723	A+B+CDT−	1
	ST92	060	A+B+CDT−	1
	ST98	037	A+B+CDT−	1
		650	A+B+CDT−	1
	ST99	070	A+B+CDT−	1
	ST102	060	A+B+CDT−	4
		066	A+B+CDT−	1
		404	A+B+CDT−	4
		595	A+B+CDT−	1
	ST103	043	A+B+CDT−	1
	ST110	499	A+B+CDT−	1
	ST139	491	A+B+CDT−	1
	ST172	060	A+B+CDT−	1
	ST234	632	A+B+CDT−	1
	ST278	666	A+B+CDT−	1
	ST286	060	A+B+CDT−	1
		545	A+B+CDT−	1
	ST512	964	A+B+CDT−	1
Clade 3	ST5	AI-56	A+B+CDT+	4
Clade 4	ST37	017	A−B+CDT−	7
	ST81	153	A−B+CDT−	3
		047	A−B+CDT−	1
		741	A−B+CDT−	1

AI-56, AI-75, AI-82, and AI-83 are ribotypes assigned by the WEBRIBO database.

The 149 strains were classified into 48 PCR-ribotypes. The most common ribotype was RT106 (17.4%, 26/149), followed by RT001 (10.1%, 15/149), RT012 (8.7%, 13/149), and RT014 (6.7%, 10/149). The prevalence of the remaining ribotypes ranged from 0.7 to 4.7% (Table 2).

MLST clades corresponded closely with toxin profiles. Clade 1 comprised almost exclusively A+B+CDT− isolates (99.2%, 132/133), Clade 3 contained only A+B+CDT+ isolates, and Clade 4 consisted solely of A−B+CDT− isolates. The most prevalent combined ST/RT designations were ST42/RT106 (17.4%, 26/149), ST3/RT001 (9.4%, 14/149), and ST54/RT012 (8.7%, 13/149) (Table 2; Figure 1).

### 3.3 Phylogenetic analysis and virulence gene carriage

#### 3.3.1 Phylogenetic structure

**Figure 2:**
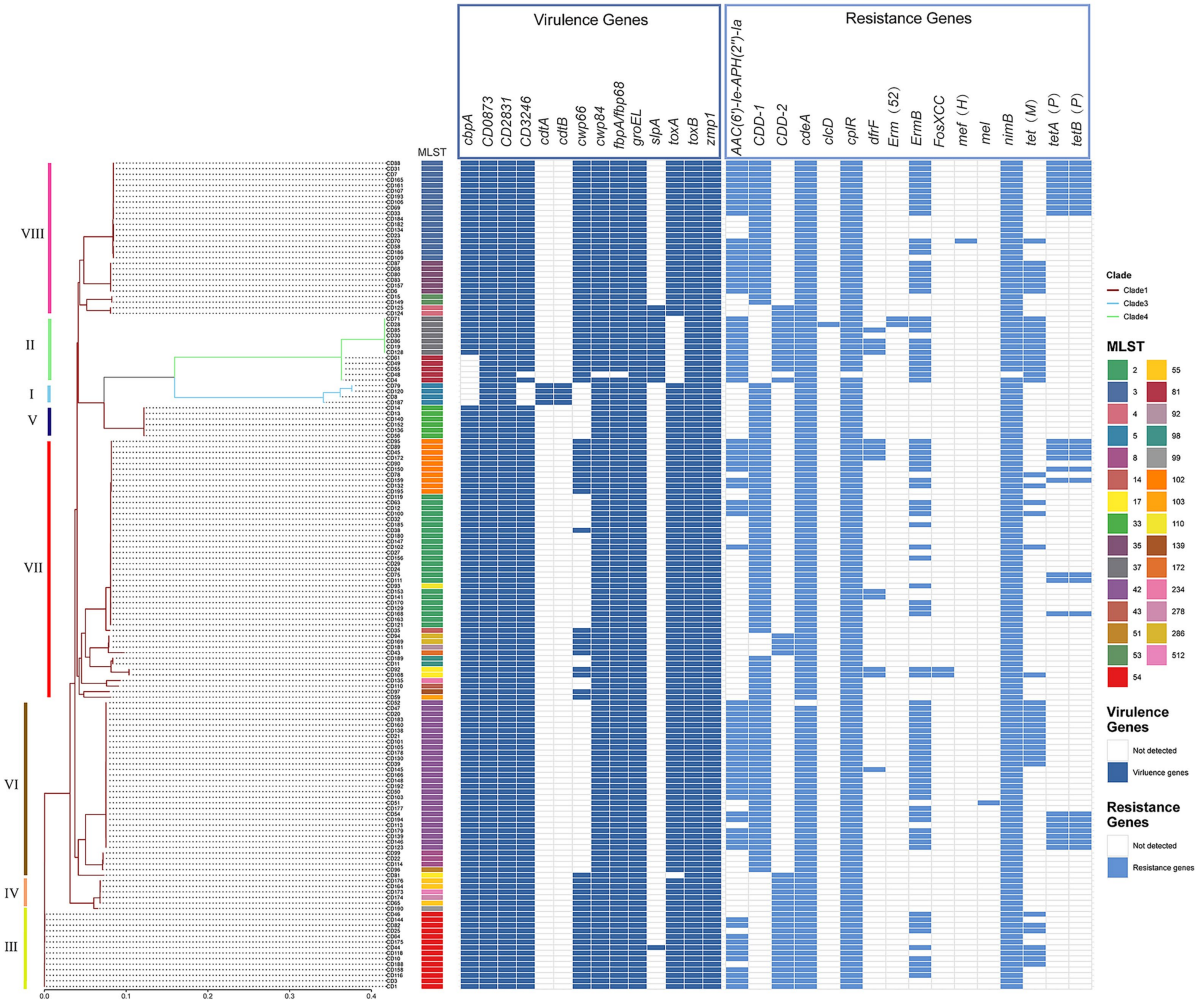
Phylogenetic tree of toxigenic *C. difficile* strains based on whole-genome sequences, annotated with strain number, MLST typing, resistance and virulence genes.

Analysis of the 149 whole-genome sequences resolved eight distinct lineages. Lineages I, III, and V each comprised a single ST: ST5, ST54, and ST33, respectively. Lineage II consisted entirely of MLST Clade 4 isolates (ST37, ST81). Lineage VII, the largest, accounted for 31.5% (47/149) of isolates and contained multiple STs. Lineage IV comprised four STs (ST55, ST512, ST278, and ST99), while lineages VI and VIII were composed predominantly of ST42 and ST3, respectively (Figure 2).

#### 3.3.2 Virulence gene detection

Fourteen virulence genes were identified by screening against the VFDB database. The adhesion genes *CD0873*, *CD2831*, and *groEL*, together with the exoenzyme gene *zmp1* and the exotoxin gene *tcdB*, were detected in all genomes. The adhesion gene *fbpA*/*fbp68* and the exoenzyme gene *cwp84* were present in 99.3% (148/149) of genomes. The adhesion gene *slpA* was detected only in ST37, ST81, ST4, and ST54 strains, whereas *cbpA* was detected in all genomes except ST81 and ST5 (Figure 2).

### 3.4 Antimicrobial susceptibility and molecular mechanisms of resistance

All *C. difficile* isolates were susceptible to vancomycin, metronidazole, and tetracycline. High resistance proportions were recorded for ciprofloxacin (84.6%, 126/149), erythromycin (56.4%, 84/149), and clindamycin (53.7%, 80/149). Lower proportions were observed for levofloxacin (16.8%, 25/149), moxifloxacin (12.1%, 18/149), meropenem (6.0%, 9/149), and rifampicin (2.7%, 4/149) (Supplementary Table S1).

Seventy-three isolates (49.0%, 73/149) were resistant to three or more antibiotics and were distributed across 10 STs; only 11 isolates were susceptible to all agents tested. Resistance patterns varied by ST. ST81 and ST37 had markedly higher resistance proportions to levofloxacin and moxifloxacin than ST2, ST54, ST102, ST33, and ST35. By contrast, ST5 and ST33 were fully susceptible to both erythromycin and clindamycin. Erythromycin- and clindamycin-resistant isolates were predominantly ST42, ST2, and ST3 (Supplementary Table S1).

Screening against the CARD identified 16 ARGs conferring resistance to aminoglycosides, β-lactams, metronidazole, fosfomycin, lincosamides, trimethoprim, macrolides, and tetracyclines. The *ermB* gene (macrolide–lincosamide–streptogramin B resistance) was detected in 97.6% (82/84) of erythromycin-resistant isolates and in 100% (80/80) of clindamycin-resistant isolates. The nitroimidazole reductase gene *nimB* was present in 99.3% (148/149) of isolates, and tetracycline resistance genes were detected at the following frequencies: *tet*(M), 29.5% (44/149); *tetA*(P), 17.4% (26/149); and *tetB*(P), 17.4% (26/149). No metronidazole- or tetracycline-resistant isolates were identified (Figure 2; Supplementary Table S1).

Mutations in the DNA gyrase genes *gyrA* and/or *gyrB* were detected in 71 isolates. Three amino acid substitutions were identified in *gyrA* (Thr82Ile, Asp205Glu, and Leu640Ser), and nine in *gyrB* (Asp426Asn, Ser366Ala, Ile139Arg, Asp426Ala, Asp426Val, Val130Ile, Arg447Lys, Asp465Asn, and Glu523Gly). Three distinct *rpoB* mutations were detected among the four rifampicin-resistant isolates: three isolates carried His502Asn, Arg505Lys, and Ile750Met concurrently, and one isolate carried only His502Asn and Arg505Lys (Supplementary Table S2).

## 4 Discussion

*C. difficile* infection has become increasingly prevalent worldwide over the past two decades and is now recognised as a leading cause of antibiotic-associated and healthcare-associated diarrhoea. In Northwest China, however, epidemiological data on CDI remain scarce, largely owing to the absence of systematic surveillance. The present study provides the first molecular epidemiological characterisation of *C. difficile* in Xi’an, integrating toxin genotyping, MLST, PCR-ribotyping, WGS-based phylogenetic analysis, virulence gene profiling, and antimicrobial susceptibility testing.

The *C. difficile* isolation proportion in this study was 8.3%, which is lower than proportions reported elsewhere in Northwest China (Li et al., 2025; Zhang et al., 2024). Toxigenic type A+B+CDT− predominated (88.6%), consistent with the global distribution. Only 13 isolates (8.7%) were identified as A−B+CDT−, a proportion considerably lower than the 23.3% previously reported in Beijing (Cheng et al., 2016). Infection with CDT-positive strains has been linked to elevated risks of mortality and recurrence (Cheng et al., 2016). Four A+B+CDT+ isolates (2.7%) were detected in our study, comparable to findings from Changsha (Meng et al., 2021). The toxin profiles were confirmed by WGS and are therefore unaffected by PCR primer-site variants; the use of orthogonal methods lends confidence that these profiles accurately reflect the circulating genotypes in Xi’an.

MLST resolved 29 STs and three phylogenetic clades among the 149 toxigenic isolates. The most prevalent genotype was ST42/RT106, followed by ST3/RT001 and ST54/RT012. This differs from the national picture: a recent survey of circulating *C. difficile* strains across China reported ST54, ST3, and ST37 as the three most common genotypes (Wen et al., 2023). Clade 1 comprised 133 isolates, almost all of which were A+B+CDT−, with a single ST17/RT532 isolate typed as A−B+CDT−. Clade 3 contained four A+B+CDT+ isolates (ST5/RTAI-56), and Clade 4 comprised 12 A−B+CDT− isolates (ST37, ST81). Core-genome SNP analysis, which offers finer resolution than MLST alone (Hakimi et al., 2025), confirmed that isolates of the same ST clustered together and revealed greater genomic diversity within Clade 1 than within Clades 3 and 4. Most genomes carried the virulence factors *CD0873*, *CD2831*, *zmp1*, *groEL*, *fbpA*/*fbp68*, *cwp84*, *CD3246*, and *cbpA*. These factors contribute, to varying degrees, to adhesion, colonisation, and biofilm formation in *C. difficile* (Dost et al., 2024). Their coordinated expression is thought to facilitate epithelial attachment and immune evasion, thereby creating conditions conducive to infection and toxin release.

Antimicrobial susceptibility testing revealed high resistance proportions to ciprofloxacin (84.6%), erythromycin (56.4%), and clindamycin (53.7%). Nearly all ciprofloxacin-resistant isolates exhibited high-level resistance (MIC ≥ 32 μg/mL). The high ciprofloxacin resistance prevalence is consistent with the known reduced susceptibility of *C. difficile* to earlier-generation fluoroquinolones. Levofloxacin resistance followed a similar pattern, whereas only 12.1% of isolates were resistant to moxifloxacin—far lower than proportions reported in Canada (Bourgault et al., 2006), likely reflecting the absence of RT027 in our collection. The Thr82Ile substitution in *gyrA* was present in every moxifloxacin-resistant isolate. The *cdeA* efflux gene was detected in nearly all isolates; the single isolate lacking *cdeA* nevertheless displayed ciprofloxacin resistance, suggesting that *cdeA* alone may not fully account for fluoroquinolone non-susceptibility (Aguilar-Zamora et al., 2021). The *ermB* gene was detected in 97.6% of erythromycin-resistant isolates and in all clindamycin-resistant isolates. All isolates remained susceptible to metronidazole and vancomycin, consistent with their continued role as first-line agents for CDI (Mutai et al., 2020). Although *tet*(M) and *tet*(W) are widely recognised as the principal tetracycline resistance determinants in *C. difficile* (Wickramage et al., 2021), no tetracycline-resistant isolates were identified in this study. Meropenem resistance was detected in nine isolates (6.0%), comparable to earlier reports from Beijing (Li et al., 2019). The Arg505Lys substitution in *rpoB* has been proposed as a key contributor to rifamycin resistance in clinical isolates (Dang et al., 2016). Consistent with this, all four rifampicin-resistant isolates (2.7%) in our cohort carried this mutation. Given the low prevalence of rifampicin resistance, rifamycin may warrant consideration as a sequential therapeutic option following vancomycin treatment for CDI.

Several limitations should be noted. First, sampling was confined to several hospitals in central Xi’an and may not fully represent the situation across the city. Second, clinical data—including underlying diseases and treatment histories—were not collected, which limits our ability to offer evidence-based recommendations for CDI diagnosis and management. Third, the small number of isolates resistant to certain antimicrobials (e.g., four rifampicin-resistant isolates) constrained the statistical power for formal genotype–phenotype correlation analysis. Future studies would benefit from a multi-center design with broader geographic coverage and prospective collection of comprehensive clinical data to enable longitudinal analysis.

In summary, this study provides the first systematic integration of molecular epidemiological data on *C. difficile* in Xi’an, combining WGS-based genotyping, virulence gene profiling, and antimicrobial resistance characterisation. The majority of toxigenic isolates belonged to type A+B+CDT−, and the predominant molecular profiles were ST42/RT106, ST3/RT001, and ST54/RT012. Although resistance to fluoroquinolones, clindamycin, and erythromycin was prevalent, all isolates remained fully susceptible to metronidazole and vancomycin. These findings point to the value of strengthening regional surveillance, reinforcing antimicrobial stewardship, and maintaining infection prevention measures to limit the spread of epidemic *C. difficile* strains.

## Data Availability

The datasets presented in this study have been deposited in the Genome Warehouse (GWH) at the National Genomics Data Center (NGDC), Beijing Institute of Genomics, Chinese Academy of Sciences, under BioProject accession number PRJCA062313, and are publicly accessible at https://ngdc.cncb.ac.cn/bioproject/browse/PRJCA062313.

## Footnotes

1
https://webribo.ages.at/

2
https://pubmlst.org/organisms/clostridioides-difficile

3
https://github.com/tseemann/abricate
